# Sex, not yeast or atrazine concentration, affects virgin adult *Drosophila melanogaster* longevity

**DOI:** 10.17912/micropub.biology.001730

**Published:** 2025-12-22

**Authors:** Rachel A. Tejiram, Pamela C. Lovejoy

**Affiliations:** 1 Department of Biology, St. Joseph's University New York

## Abstract

In *Drosophila melanogaster* , the herbicide atrazine is known to alter longevity, accelerate development time, and cause modifications in protein production and gene expression related to oxidative stress. A low protein diet can affect fecundity and increase lifespan in flies. The present study investigated if and how different concentrations of dietary yeast, the main protein source for lab-reared flies, affect the lifespan of *D. melanogaster* exposed to atrazine. Atrazine exposure and yeast concentration did not affect adult longevity; however, there was a strong sex effect in that males displayed greater survival than females.

**Figure 1. The effect of yeast and atrazine concentrations on the longevity of D. melanogaster:**
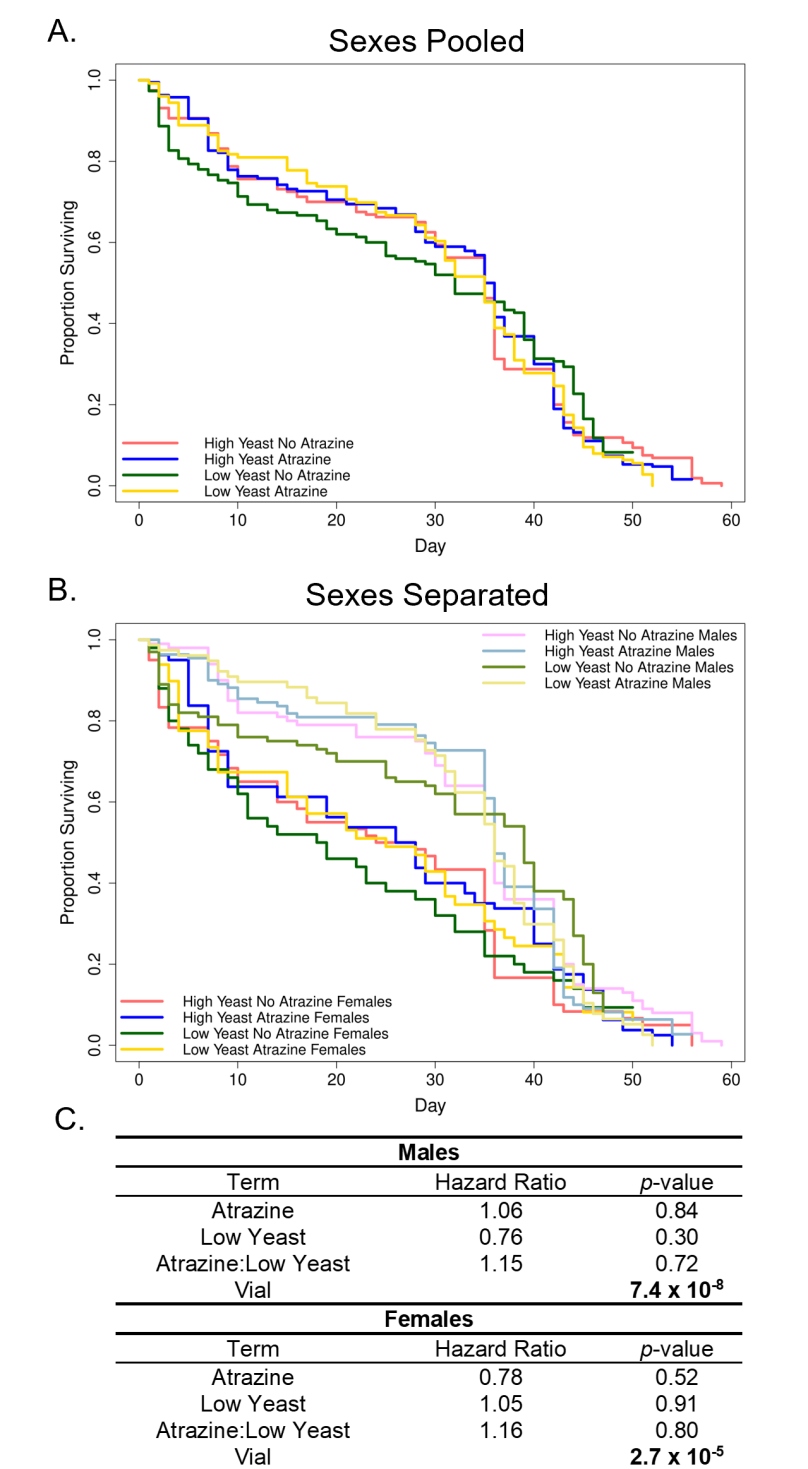
*Drosophila melanogaster* were reared on one of four food treatments as described in Table 1: high yeast with no atrazine (control; HYNA), high yeast with 2 ppM atrazine (HYA), low yeast with no atrazine (LYNA), or low yeast with 2 ppM atrazine (LYA). **(A.) Yeast and atrazine concentrations do not affect survival.** There were no differences in survival between any of the four food treatments ( *p* > 0.05). Lines represent the proportion of surviving virgin adult *D. melanogaster* with sexes pooled. HYNA is dark red (n=160), HYA is dark blue (n=190), LYNA is dark green (n=150), and LYA is dark yellow (n=126). **(B.) Sex affects survival.** There was a significant difference in survival between males and females ( *p* < 0.001). Lines represent the proportion of surviving virgin adult male and female *D. melanogaster* . HYNA Females is dark red (n=60), HYA Females is dark blue (n=80), LYNA Females is dark green (n=50), and LYA Females is dark yellow (n=49). HYNA Males is light red (n=100), HYA Males is light blue (n=110), LYNA Males is light green (n=100), and LYA Males is light yellow (n=77). **(C.) Cox regression analysis for longevity.** Cox regression terms are shown along with their hazard ratios and statistical significance. A hazard ratio greater than 1 implies that the term decreases survival while a hazard ratio less than 1 implies that the term increases survival. Bold *p* – values signify statistical significance.

## Description

Atrazine, a triazine herbicide, has been designed to repress the growth of weeds in agricultural and recreational settings (Rinsky et al., 2012; Stradtman and Freeman, 2021). Atrazine has been shown to have negative effects on both vertebrate and invertebrate non-target organisms, including causing reduced numbers of viable eggs in male African clawed frogs (Hayes et al., 2010), liver cell abnormalities in rats (Campos-Pereira et al., 2012), and disruption of neurotransmitter signals in honeybee hearts (Papaefthimiou et al., 2003). Additionally, *Drosophila melanogaster* has been shown to be negatively impacted by atrazine. In *D. melanogaster* , atrazine alters the expression of proteins linked to energy production, specifically respiration and glycolysis, and those related to oxidative stress (Thornton et al., 2010). Furthermore, one study showed that flies subjected to atrazine exposure exhibit significant decreases in survival (Marcus and Fiumera, 2016), which could be because of elevated reactive oxygen species (ROS) levels caused by atrazine (Figueira et al., 2017). Another study that examined virgin *D. melanogaster* found that atrazine increased longevity (Lovejoy & Fiumera, 2019).

In addition to environmental stressors like atrazine, diet can affect organismal fitness. Diet nutritional value has been shown to affect *D. melanogaster* survival rate (Tatar et al., 2014; Meshrif and Elkholy, 2015). Ratios of nutrients, specifically protein (P) and carbohydrate (C), affect fecundity and longevity (Lee et al., 2008; Kim et al., 2020). For example, *Drosophila* that consume protein-to-carbohydrate (P:C) diets with ratios between 1:10 and 1:20 have been shown to have maximized longevity when compared to other ratios such as 1:1, 1:2, 1:4, 1:8, and 1:32 (Bruce et al., 2013). This is in agreement with another study that identified 1:16 as the optimal P:C ratio (Lee et al., 2008).

*Drosophila melanogaster* has often been utilized in investigations of survival upon exposure to various stressors (Clark and Fucito, 1998; Bushey et al., 2010; Mockett and Matsumoto, 2014; Weisman et al., 2014, among others). As exposure to multiple stressors can sometimes have increased or synergistic effects (Thiruchelvam et al., 2000; Anderson and Lydy, 2002; Patel et al., 2006; Wang et al., 2016), *D. melanogaster* can also be useful in evaluating the effects of exposure to multiple stressors. In the present study, we investigated the effects of atrazine exposure and diet deficiency on longevity. To evaluate the effects of these factors in combination, *D. melanogaster* of the Oregon-R strain were reared on one of four different diets (Table 1), which were high yeast with no atrazine (control; HYNA), high yeast with 2 ppM atrazine (HYA), low yeast with no atrazine (LYNA), or low yeast with 2 ppM atrazine (LYA). Due to the use of multiple stressors in this study, the low yeast treatment contained 25% of the typical amount of brewer’s yeast/ *S. cerevisiae* that is used in our fly food (a 1:4 P:C ratio). It was hypothesized that *D. melanogaster* raised on a high yeast concentration as opposed to a low yeast concentration will show increased longevity when atrazine is present. We predicted that, over the course of 60 days, the proportion of surviving flies raised on the LYNA diet would show forth a statistically significant decrease compared to the HYNA diet.

Our results show that male and female *D. melanogaster* longevity was not affected by atrazine exposure ( Figure 1A- C; *p* = 0.84 in males and *p* = 0.52 in females). Interestingly, this is contrary to the findings of other studies that have examined similar factors. One such study has shown that 2 ppM atrazine exposure significantly decreases *D. melanogaster* longevity (Marcus and Fiumera, 2016). This effect may occur because atrazine influences oxidative stress in that ROS are elevated (Figueira et al., 2017). Heightened levels of ROS have been known to influence trade-offs with survivability and reproduction (Dowling and Simmons, 2009; Lang et al., 2021). Marcus and Fiumera used mated flies to measure the effects of atrazine, whereas the present study used virgin flies. This alteration may explain the difference in effects between studies, as mating has been shown to augment vulnerability to oxidative stress (Rush et al., 2007) which may then be compounded by the oxidative stress caused by atrazine. As previously mentioned, however, another study showed that atrazine increased the longevity of virgin *D. melanogaster* (Lovejoy and Fiumera, 2019). Atrazine seems like it may interact with other factors to affect longevity and its effects should be studied further.

Diet, specifically P:C ratio, has been known to affect longevity in *D. melanogaster* (Lee et al., 2008; Lushchak et al., 2012; Bruce et al., 2013; Lee, 2015). Other research examining dietary restriction through yeast dilution also reported alterations to longevity (Grandison et al., 2009). In the current study, we altered P:C ratios through changes in dietary yeast concentration, but we did not find that this affected adult longevity ( Figure 1A- C; *p* = 0.30 in males and *p* = 0.91 in females). The P:C ratios of both high yeast diets (HYNA and HYA) were 1:1 while the P:C ratios of both low yeast diets (LYNA and LYA) were 1:4. All diets used in this study contained more protein relative to carbohydrate than those found to produce optimal longevity in some of the previously mentioned studies (eg. between 1:10 and 1:20 ratios in Bruce et al., 2013 and 1:16 in Lee et al., 2008). This may explain why there were no observable effects of diet on adult longevity.

Sex had a strong effect on adult longevity in that males displayed greater survival than females across all treatments ( Figure 1B ; *p* < 0.001). This is consistent with Lin et al. 2023, a study that utilized males and females from 15 strains of *D. melanogaster* and found that when the strains, including the Oregon-R strain, were assessed altogether, males lived longer than females (Lin et al., 2023). Nevertheless, other studies have shown that males do not always live longer than females (Niveditha et al., 2017; Brown et al., 2020) and that this trait may vary by strain. Hence, sex differences in individual strains are clear in the literature but should be further researched for a more complete understanding of longevity.

Taken together, our results show that adult longevity is significantly affected by sex, but not atrazine exposure or yeast concentration. All in all, sex, developmental stages, nutrition, and the presence of stressors in environments should be considered in future experiments involving longevity.

## Methods

***Drosophila melanogaster* strains and media**

*Drosophila melanogaster* from the wild-type Oregon-R stock were utilized in this experiment. Flies were maintained on a 12-hour light/dark cycle in a humidified incubator at 25°C. Flies were raised on one of four food treatments (Table 1) that are based on a standard agar-dextrose-yeast media (McGraw et al., 2007). LYNA and LYA foods contained yeast that was 25% of the amount of yeast in the HYNA and HYA foods. HYA and LYA foods contained atrazine at a concentration of 2 ppM. This was accomplished by diluting 20 ppM atrazine water (the highest concentration that atrazine can be dissolved in water) during food preparation as shown in Table 1. A concentration of 2 ppM was chosen for this study because it has been shown to significantly affect a range of different traits in *D. melanogaster* (Marcus and Fiumera, 2016).

Table 1:&nbsp; Sample recipe to make 500 mL of each food treatment.

**Table:** 

**Food Type**	**High Yeast,** **No Atrazine** **(HYNA; Control Diet)**	**High Yeast,** **Atrazine (HYA)**	**Low Yeast,** **No Atrazine (LYNA)**	**Low Yeast,** **Atrazine (LYA)**
** dH _2_ O **	500 mL	450 mL	500 mL	450 mL
**20 ppM** **Atrazine**	0 mL	50 mL	0 mL	50 mL
**Agar**	5 g	5 g&nbsp;	5 g	5 g
**Dextrose**	41.66 g	41.66 g	41.66 g	41.66 g
** Brewer’s yeas *t (S. cerevisiae)***	41.66 g	41.66 g	10.415 g	10.415 g
**8.3% Phosphoric Acid**	2.5 mL	2.5 mL	2.5 mL	2.5 mL
**83.6% Propionic Acid**	2.5 mL	2.5 mL	2.5 mL	2.5 mL

&nbsp;

**Longevity**

Adult flies, which had previously been maintained on control food, were allowed to lay eggs on one of the four food treatments for several days and were then discarded. Male and female virgin offspring were collected and separated within eight hours of emergence and transferred to vials containing the same food that they developed on. (Note that the flies in both low yeast treatments needed an additional week to emerge as adults.) Flies were allocated into vials in low-density, single-sex groups of ten when possible; however, some vials, specifically with the LYA diet, contained less than ten flies, as they were more difficult to collect. Only flies that were collected on the same day were housed together in a vial. The number of vials per treatment group per sex included: HYNA Females (n=6), HYNA Males (n=10), HYA Females (n=8), HYA Males (n=11), LYNA Females (n=5), LYNA Males (n=10), LYA Females (n=7), and LYA Males (n=10). The collection of all groups continued until there were approximately 50 males and females for each treatment group, which resulted in a larger sample size of male flies compared to female flies. Longevity was measured starting from the day of eclosion. Dead flies were recorded twice per week. Additionally, flies were transferred into new vials with their respective food treatments once a week.

**Statistical Analysis**

Statistical analysis was performed as a Cox proportional hazards regression using R version 4.1.1 with the *coxph* function from the *survival* package (Therneau, 2023). Because of a strong effect of sex in the initial pooled model ( *p* < 0.001), statistical analysis was performed separately for males and females. The models each contained an interaction term for atrazine and yeast, and also included vial as a random factor using the *frailty* function in the *survival* package ( Figure 1C ).

## Reagents

**Table:** 

**Reagent:**	**Obtained from:**
*D. melanogaster* strain Oregon-R	Bloomington *Drosophila* Stock Center (Stock #5)
Atrazine	Sigma Aldrich; 45330
Agar	Genesee Scientific; 66-104
Dextrose	BSG craft brewing; AZZZ3305
Brewer’s yeast ( *S. cerevisiae* )	MP Biomedicals; 02903312-CF
